# Effects of allicin on human Simpson-Golabi-Behmel syndrome cells in mediating browning phenotype

**DOI:** 10.3389/fendo.2023.1141303

**Published:** 2023-03-01

**Authors:** Uzair Ali, Martin Wabitsch, Daniel Tews, Monica Colitti

**Affiliations:** ^1^ Department of Agricultural, Food, Environmental and Animal Sciences, University of Udine, Udine, Italy; ^2^ Division of Pediatric Endocrinology and Diabetes, Department of Pediatrics and Adolescent Medicine, Ulm University Medical Center, Ulm, Germany

**Keywords:** SGBS cells, allicin, lipid droplets, mitochondria, thermogenesis

## Abstract

**Introduction:**

Obesity is a major health problem because it is associated with increased risk of cardiovascular disease, diabetes, hypertension, and some cancers. Strategies to prevent or reduce obesity focus mainly on the possible effects of natural compounds that can induce a phenotype of browning adipocytes capable of releasing energy in the form of heat. Allicin, a bioactive component of garlic with numerous pharmacological functions, is known to stimulate energy metabolism.

**Methods:**

In the present study, the effects of allicin on human Simpson-Golabi-Behmel Syndrome (SGBS) cells were investigated by quantifying the dynamics of lipid droplets (LDs) and mitochondria, as well as transcriptomic changes after six days of differentiation.

**Results:**

Allicin significantly promoted the reduction in the surface area and size of LDs, leading to the formation of multilocular adipocytes, which was confirmed by the upregulation of genes related to lipolysis. The increase in the number and decrease in the mean aspect ratio of mitochondria in allicin-treated cells indicate a shift in mitochondrial dynamics toward fission. The structural results are confirmed by transcriptomic analysis showing a significant arrangement of gene expression associated with beige adipocytes, in particular increased expression of T-box transcription factor 1 (TBX1), uncoupling protein 1 (UCP1), PPARG coactivator 1 alpha (PPARGC1A), peroxisome proliferator-activated receptor alpha (PPARA), and OXPHOS-related genes. The most promising targets are nuclear genes such as retinoid X receptor alpha (RXRA), retinoid X receptor gamma (RXRG), nuclear receptor subfamily 1 group H member 3 (NR1H3), nuclear receptor subfamily 1 group H member 4 (NR1H4), PPARA, and oestrogen receptor 1 (ESR1).

**Discussion:**

Transcriptomic data and the network pharmacology-based approach revealed that genes and potential targets of allicin are involved in ligand-activated transcription factor activity, intracellular receptor signalling, regulation of cold-induced thermogenesis, and positive regulation of lipid metabolism. The present study highlights the potential role of allicin in triggering browning in human SGBS cells by affecting the LD dynamics, mitochondrial morphology, and expression of brown marker genes. Understanding the potential targets through which allicin promotes this effect may reveal the underlying signalling pathways and support these findings.

## Introduction

Obesity is a complex multifactorial disease that presents a risk of death as it is associated with many noncommunicable diseases such as cardiovascular diseases, type 2 diabetes, and cancer. Since the discovery of brown adipose tissue (BAT) in the adult human body and its ability to dissipate energy, it has been of particular interest to exploit the activity of BAT as a therapeutic option to counteract obesity. In addition, the formation of thermogenic or beige adipocytes in white adipose tissue (e.g., adipocyte browning) may represent another option to increase energy expenditure (1). In both brown and beige adipocytes oxidative phosphorylation is uncoupled from ATP production, which is due to up-regulation of uncoupling protein-1 (UCP1) (2). To date, *in vitro* and *in vivo* studies have identified a considerable number of browning agents, such as capsaicin, resveratrol, caffeine, and fucoxanthin (3, 4). Garlic (*Allium sativum* L.) is a popular species rich in organosulfur compounds that are useful for medicinal purposes. When garlic is chopped or crushed, alliin is released and then hydrolyzed into allicin by allicinase. Allicin *in vitro* breaks down into a variety of fat-soluble organosulfur compounds, including diallyl trisulfide (DATS), diallyl disulfide (DADS), and diallyl sulfide (DAS) (5–7). The high permeability of allicin through cell membranes and rapid reaction with free thiol groups promote its diverse biological and therapeutic functions (8). Allicin is known for its antibacterial, antifungal, and antiparasitic activities (9), as well as its anticarcinogenic (10, 11) and anti-inflammatory functions (12, 13). Allicin has also been shown to suppress cholesterol biosynthesis by inhibiting squalene monooxygenase and acetyl-CoA synthetase (14). Methanolic extract of black garlic containing alliin, upregulated the expression of genes related to adipokines, lipolysis, and fatty acid oxidation in adipose tissue of rats fed a high-fat diet (15).

Recently, allicin was reported to promote browning in differentiated 3T3-L1 adipocytes and white inguinal adipose tissue of mice through extracellular signal regulated kinase 1/2 (ERK1/2) and KLF Transcription Factor 15 (KLF15) pathways, which stimulates the expression of UCP1 through interaction with its promoter (16). It has also been suggested that the Sirt1-PGC1α-Tfam pathway plays a role in promoting allicin-mediated BAT activity (17).

Although several mouse cell lines are available to understand the adipogenic and thermogenic regulatory networks *in vitro*, human cell lines are of interest to explore the molecular mechanism of browning and to identify potential dietary supplements and nutraceuticals that could induce browning. The Simpson-Golabi-Behmel Syndrome (SGBS) cell strain is commonly used as a model for the differentiation of human white adipocytes (18). These cells retain their differentiation ability up to several generations when provided with the appropriate adipogenic differentiation medium. Based on the effect of rosiglitazone (19), a browning phenotype was observed in SGBS cells during differentiation, and RNA sequencing revealed an increase in genes involved in extracellular matrix organization and oxidative stress that may regulate adaptive thermogenesis, with an increased percentage of brown phenotype, confirming that differentiated SGBS cells gradually acquire BAT-like function from day 4 to day 10 (20).

After stimulation of browning, the formation of micro lipid droplets (LDs) has been demonstrated in response to lipolytic release of fatty acids (21). This enables efficient intracellular lipolysis from the LD surface and subsequent promotion of free fatty acid transport to mitochondria for β-oxidation in BAT (22). Consistent with this property, both cold exposure and adrenergic stimulation induce rapid mitochondrial fragmentation, which synergistically promote uncoupling and thus heat production (23).

Using RNAseq and quantifying the dynamics of LDs and mitochondrial morphology, the current study aims to evaluate the browning effect of allicin *in vitro* using the SGBS cell strain as a human primary adipocyte model in comparison to the control and cells treated with dibutyryl cAMP sodium salt (cAMP) as a positive control. To clarify the potential browning effect of allicin, a network pharmacology strategy was also performed based on the identification of potential targets.

## Materials and methods

### Chemicals and culture media

Dulbecco’s modified Eagle medium (DMEM)/F-12 medium (1:1) enriched with L-glutamine and 15 mM 4-(2-hydroxyethyl)-1-piperazineethanesulfonic acid (HEPES), fetal bovine serum (FBS) and penicillin streptomycin solution were purchased from Gibco by Life Technologies (Thermo Fisher Scientific Inc., Waltham, Massachusetts). TRIzol reagent, PureLink™ RNA Mini Kit and SuperScript™ III one-step RT-PCR system with Platinum™ *Taq* DNA polymerase were purchased from Invitrogen (Thermo Fisher Scientific Inc., Waltham, Massachusetts). Rosiglitazone was purchased from Cayman Chemical (Ann Arbor, Michigan). Allicin was purchased from Solarbio Life Sciences^®^ (Beijing, China).

All other chemicals used in the experiment and not listed above were purchased from Sigma-Aldrich (Darmstadt, Germany).

### Cell culture and treatments

Human SGBS cells were grown Dulbecco’s Modified Eagle Medium Nutrient Mixture F-12 (DMEM/F-12) supplemented with 10% fetal bovine serum (FBS), 3.3 mM biotin, 1.7 mM panthotenate and 1% penicillin/streptomycin solution, at 37°C, 5% CO_2_ and 95% relative humidity. Cells were platted in Petri dishes (100mm) in duplicate. Once the cells reached approximately 90% confluence, differentiation was induced by feeding the cells with serum-free growth medium supplemented with 10 µg/ml transferrin, 0.2 nM triiodothyronine (T_3_), 250 nM hydroxycortisone, 20 nM human insulin, 25 nM dexamethasone, 250 µM 3-isobutyl-1-methylxanthine (IBMX) and 2 µM rosiglitazone (day 0 of differentiation). After 4 days, the differentiation medium was replaced with maintenance medium composed by serum-free growth medium supplemented with 10 µg/ml transferrin, 0.2 nM T_3_, 250 nM hydroxycortisone and 20 nM human insulin. Fresh maintenance medium was added every 2 days.

**Figure 1:**
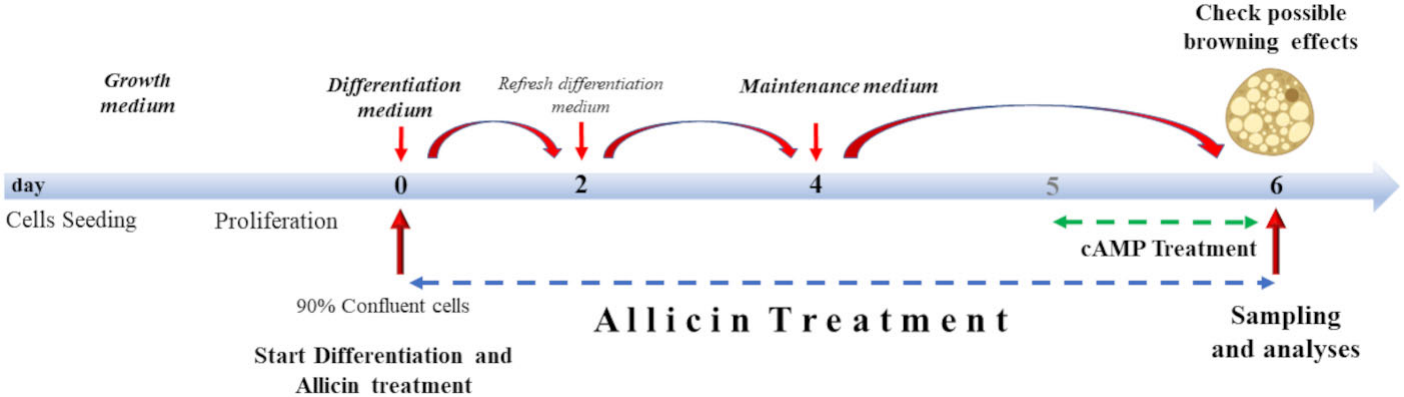
Cell culture treatment protocol. Cells were grown to 90% confluence. Differentiation lasted 6 days supplemented with allicin treatment. From day 4, cells were incubated with allicin in maintenance medium. Cells were incubated with cAMP for 24 hours (from day 5 to day 6 of differentiation). Sampling and analyses were performed on day 6.

Treatment with allicin began on day 0 of differentiation (D0), and continued until analysis on day six (D06) of differentiation (**Figure 1**). Allicin concentrations of 5, 12.5, 25, and 50 µM were tested. Prior to treatment, allicin was diluted in dimethyl sulfoxide (DMSO) and a stock solution was prepared. Stock solutions were prepared so that the volume of DMSO in the treatment medium did not exceed 0.5%. Control cells (CTRL) were incubated with the same volume of 0.5% DMSO in the differentiation medium for 6 days. As a positive control (cAMP), SGBS cells were treated with 500 µM dibutyryl cAMP sodium salt (a cyclic nucleotide derivative that mimics endogenous cAMP) for 24 hours before the sixth day of differentiation (24) (**Figure 1**).

### Cell viability assay

Cell viability was determined using the 3-(4,5-dimethylthiazol-2-yl)-2,5-diphenyltetrazolium bromide (MTT) assay. Cells were plated in a 96-well plate and treated with 5.0, 12.5, 25.0, and 50 µM allicin (25). Before incubation with 5 mg/mL MTT in HBSS, cells were rinsed with 1X phosphate buffer saline (PBS) 1X. Incubation with the MTT solution was performed at 37°C for 4 hours. The resulting formazan was dissolved in dimethyl sulfoxide (DMSO) and incubated overnight (O/N) at 37°C. Optical density was used as an indicator of cell viability and was measured at 590 nm.

### BODIPY staining and confocal imaging

Cells for BODIPY™ staining and subsequent confocal imaging were cultured on ibiTreat 8-well μ-slides (Ibidi GmbH, Planegg/Martinsried, Germany). Cells were fixed in a 2% formalin solution diluted in PBS 1X at room temperature (RT) for 15 minutes. Subsequently, after washing three times in PBS 1X, the cells were incubated in a solution of BODIPY™ 493/503 in PBS 1X to fluorescently label the lipid droplets. Incubation was performed for 45 minutes in the dark at RT. The slides were then washed three times in PBS 1X.

Fluorescence images were acquired using a Leica SP8 confocal microscope (Leica Microsystems Srl, Milan, Italy) and LAS X 3.1.5.16308 software. Slides were viewed with the HCX PL APO lambda blue 63x/1.40 OIL objective. DAPI fluorescence was detected with a 405 diode laser (410/480 nm), while BODIPY fluorescence was detected with a white light laser (503/588 nm). Images were acquired using a photomultiplier tube (PMT) that allowed point scanning of the region of interest (ROI) with the selected laser and produced 1024 × 1024 px images.

### Morphology of LDs

The MRI_Lipid Droplets Tool (http://dev.mri.cnrs.fr/projects/imagej-macros/wiki/LipidDroplets_Tool), a macro in the ImageJ 1.50b software (http://rsb.info.nih.gov/ij/), was used to measure LD area (26). Individual cells were defined by regions of interest (ROIs) and images were analyzed as previously described (27). For each cell, the LD area (in μm^2^), maximum Feret diameter (MFD, in μm), and integrated optical density (IOD, dimensionless) were measured. The MDF is used as a measure of the diameter of irregularly shaped objects, whereas the IOD is related to both triglyceride accumulation and the size of LDs (28).

### MitoTracker® staining

SGBS cells cultured on ibiTreat 8-well μ-slides (Ibidi GmbH, Germany) were incubated at 37°C with 100 nM MitoTracker^®^ Orange CMTMRos (Thermo Fisher Scientific, USA) for 30 minutes. The stained cells were washed with PBS 1X and fixed with 2% formalin at RT for 15 minutes. After fixation, cells were rinsed three times with PBS 1X, mounted in DAPI-containing mounting medium (Cayman Chemical Company, USA), and imaged using a Leica SP8 confocal microscope (Leica Microsystems, Germany) and LAS X 3.1.5.16308 software. Slides were viewed with the HCX PL APO lambda blue 63x/1.40 OIL objective. DAPI fluorescence was detected with a 405 diode laser (410/480 nm), while MitoTracker^®^ fluorescence was detected with a white light laser (550/605 nm). Images were acquired using a photomultiplier tube (PMT) that allowed point-by-point scanning of the region of interest (ROI) with the selected laser and produced images with a resolution of 1024 x 1024 px.

### Mitochondrial morphology analyses

To quantify mitochondrial morphology on standard confocal fluorescence microscopy images of CTRL, ALLI, and cAMP-treated cells, the Mitochondrial Analyzer based on adaptive thresholding and the ImageJ/Fiji open-source image analysis platform were used (29). Scale was set for magnification and the global check box in the Set Scale dialog box was selected. After 2D threshold optimization, the images were thresholded with a block size of 1,350µm and a C-value of 5. Subsequently, the images were also processed using the MiNa (30) and Micro2P (31) tools.

The Mitochondria Analyzer tool was used to measure counts (number of mitochondria in the image), total area (sum of the area of all mitochondria in the image), mean area (total area/mitochondria number), total perimeter (sum of perimeter of all mitochondria in the image), mean perimeter (total perimeter/mitochondria number), mean aspect ratio (shape descriptor measuring elongation), and mean form factor (shape descriptor measuring round to filamentous shape). In addition, parameters describing the connectivity of the mitochondrial network were calculated, including the number of branches, the total length of branches, the mean length of branches, the branch junctions, the end points of branches, and the mean diameter of branches. The branches consist of point-shaped objects without branching junctions and minimal length, long single tubular objects without branching junctions, but the highest branch length and complex objects with multiple branches and junctions. The number of branches, total branch length, branch junctions and branch end points were also expressed as normalization to either the number of mitochondria or total area (29).

The MiNa tool, a macro of the ImageJ1.53o software (http://rsb.info.nih.gov/ij/), was also used to quantify mitochondrial morphology (30). Threshold images were processed using the Tophat option (32), as was the MiNa interface. The macro detected ‘individual’ mitochondrial structures in a skeletonized image, such as punctate, rod-shaped, and large/round structures without branching, and ‘networks’ identified as mitochondrial structures with a single node and three branches. All parameters were used in the discriminant analysis. Among the nine parameters calculated by MiNa, the number of individuals (punctate, rod-shaped, and large/round mitochondria), the number of networks (objects with at least one branch), and the mean rod/branch length, which refers to the average length of all mitochondrial rods/branches, were considered for statistical comparisons. Other parameters such as the mean number of branches per network, i.e., the mean number of mitochondrial branches per network, the mean length of branches, and the mitochondrial footprint, which refers to the total area of mitochondria, were included in the discriminant analysis.

Mitochondria were also analyzed and classified using MicroP software, a useful tool validated in CHO-K1 cells (31). The software classifies six morphological types of mitochondria, such as small globules, round-shaped mitochondria, that may have arisen by fission; large globules with a larger area; simple tubules, i.e. straight, elongated mitochondria without branches; twisted tubules, elongated tubular mitochondria with a non-linear development; donuts, like elongated tubules mitochondria but with fused ends; branched tubules, complex interconnected mitochondria with a network-like structure. On each image, the total number of mitochondria and their area were calculated as the ratio of mitochondria and area for each subtype in the different SGBS cells treated. These data with the number of objects and total area were used for discriminant analysis.

### RNA extraction and sequencing

The experiment was set up with 2 biological replicates for the 3 experimental conditions.

After removing the culture medium from the Petri dishes, 1ml/10cm^2^ of TRIzol reagent was added to each plate and repeatedly pipetted to induce a severe breakdown of the cell structures. These samples were immediately processed further using the PureLink™ RNA Mini Kit according to the manufacturer’s instructions.

The concentration of extracted total RNA was quantified using a spectrophotometer (NanoDrop 1000 Spectrophotometer, ThermoScientific, Wilmington, Delaware), and the purity of the RNA samples ranged from 1.8 to 1.9. RNA integrity was assessed by observing the 18S and 28S ribosomal bands after electrophoresis on 1% agarose gel, in the presence of GelRed. β-actin expression was used as an internal control, and confirmed the complete integrity of the RNA.

The purified total RNA was subjected to deep sequencing analysis. First, the isolated RNA was quantified using Agilent Bioanalyzer 2100 with the RNA integrity number (RIN) greater than 8.0 before sequencing using Illumina Genome Analyzer (GA). Generally, 2-4 ug of the total RNA was used for library construction. Total RNA was reverse transcribed into double-stranded cDNA, digested with NlaIII and ligated to an Illumina specific adapter containing a recognition site of MmeI. After MmeI digestion, a second Illumina adapter, containing a 2-bp degenerate 3’ overhang was ligated. The obtained sequences were aligned on GRCh38 human genome (https://www.ncbi.nlm.nih.gov/assembly/GCF_000001405.39) using STAR software (33).

### Data processing

Raw data were uploaded to the R package (v0.92) Differential Expression and Pathway analysis (iDEP951) that is a web-based tool available at http://bioinformatics.sdstate.edu/idep/(34, 35). In the pre-processing step, genes expressed at very low levels across samples were filtered out, and genes expressed at a minimum of 0.5 counts per million (CPM) in a library were further analyzed. To reduce variability and normalizecount data, EdgeR log2(CPM+c) was chosen with pseudocount c = 4 transformation,. Next, the DESeq2 package in the R language was used to identify differentially expressed genes (DEG) between ALLI_ cAMP, ALLI_CTRL and cAMP_CTRL using a of false discovery rate (FDR) threshold ≤ 0.05 and fold-change > |1.0|. Heatmaps, principal component analysis (PCA), k-means cluster and enrichment analyses were also performed in iDEP951.

Gene set enrichment analysis to determine the shared biological functions of differentially regulated genes based on significant GO terms (36), Kyoto Encyclopedia of Genes and Genomes (KEGG) pathway (37) and TF. target.TRED analyses were performed (38).

Venn diagrams were created by web tool available at http://bioinformatics.psb.ugent.be/webtools/Venn/.

### Protein-protein interaction Network construction and hub genes analysis

On the basis of the online tool Search Tool for the Retrieval of Interacting Genes (STRING; https://string-db.org/), PPI networks of the up regulated and the down regulated DEGs in each comparison were generated with a confidence level of ≥ 0.4, and the PPI network was visualized using Cytoscape software (version 3.9.1, https://cytoscape.org/). Then, the PPI networks of DEGs in each comparison were analyzed using the Cytoscape CytoHubba plugin to select the top 10 hub nodes according to the Degree algorithm (39). The Molecular Complex Detection (MCODE) plug-in (40) in the Cytoscape suite was used to examine the significant modules in the PPI network of overlapping DEGs that are the target ofshared TF networks between comparisons. Degree cutoff = 2, K-core = 2, and node score cutoff = 0.2 were set as options. Enrichment analysis of DEGs in modules with a score ≥ 5 was then performed.

### PROFAT webtool analysis

Estimation of the proportion of brown adipocytes in each sample was analyzed based on read counts using the PROFAT tool, which automatically performs hierarchical cluster analysis to predict the browning capacity of mouse and human RNA-seq datasets (41).

### Targets prediction of allicin, DAS, DADS, DATS

To identify a larger number of potential targets, PharmMapper (http://www.lilab-ecust.cn/pharmmapper/; 42, 43), the similarity ensemble approach (SEA, https://sea.bkslab.org/), the STITCH database (http://stitch.embl.de/; 44), Swiss Target Prediction (http://www.swisstargetprediction.ch/; 45), and GeneCard (https://www.genecards.org/) were used. The 2D structure and canonical SMILES of allicin (CID_65036), diallyl sulphide (CID_11617), diallyl disulfide (CID_16590), and diallyl trisulfide (CID_16315) were obtained from the PubChem database (https://pubchem.ncbi.nlm.nih.gov/). The sdf files were uploaded to the PharmMapper server, and the search was started using the maximum generated conformations of 300 by selecting the option ‘Human Protein Targets Only (v2010, 2241)’ and the default value of 300 for the number of reserved matching targets. for the other parameters, the ‘default mode’ was selected. Canonical SMILES were uploaded to the other tools. The predicted targets were entered into the UniProt database (https://www.uniprot.org/) with the species set to *Homo sapiens* to determine their gene IDs. A Venn diagram was used to find common targets among the allicin compounds. Genes related to ‘adipocyte’, ‘browning’, ‘non shivering thermogenesis’, ‘cold-induced thermogenesis’, ‘brown adipose thermogenesis,’ and ‘adaptive thermogenesis’ were downloaded from GeneCard, and the intersection of the targets was determined using the Venn tool. The resulting common genes associated with browning and adipocytes were then crossed with common putative targets of allicin, and a Venn diagram was generated. Subsequently, the overlapping targets were uploaded to GeneMANIA (https://genemania.org/) (46) to perform functional gene analysis and generate a PPI network.

CytoNCA, another Cytoscape plugin, was applied to the network to perform topological analysis evaluating the centrality measures of the network (47). Then, the Cytoscape intersectional merge function was used to isolate the PPI subnetworks. Key node functions were determined by analyzing GO terms, KEGG and Reactome pathways.

By entering the screened key nodes into the online tool VarElect (48), the correlation between nodes and ‘cold induced thermogenesis’ was investigated.

### Statistical analysis

All measurement results are given as means ± SD and were analyzed with XLSTAT (49). Measurements of LD area surface/cell, MFD/cell, and IOD/cell obtained from 15 biological replicates were compared along with Mitochondrial Analyzer, MiNa, and Micro2P results using the Kruskal-Wallis statistical test, followed by pairwise comparisons using the Mann-Whitney approach with Bonferroni correction (*p <* 0.0167).

All Mitochondrial Analyzer and MiNa parameters, as well as ratios of parameters obtained with the Micro2P tool, were calculated together to perform a canonical discriminant analysis (DA) that integrates morphological mitochondrial parameters into a single multivariate model with the aim of maximizing differences between treatments and calculating the best discriminant components between treatments (49).

## Results

### Cell viability

To investigate possible adverse effects of allicin, a viability assay was used to investigate possible adverse effects of ALLI extract on SGBS cells treated with doses of 5, 12.5, 25, and 50 µM. In particular, the analysis showed that the viability of cells treated with ALLI extract decreased significantly (*p <* 0.001) in a dose-dependent manner (**Figure S1**), with the 50 µM dose always significantly different from the other doses. Nevertheless, viability remained above 85% up to 25 µM and 12.5 µM ALLI and did not differ from 5 and 25 µM. Based on these findings, 12.5 µM ALLI was selected for further experiments.

### Allicin treatment affects the number of lipid droplets and their maximum diameter

**Figure 2:**
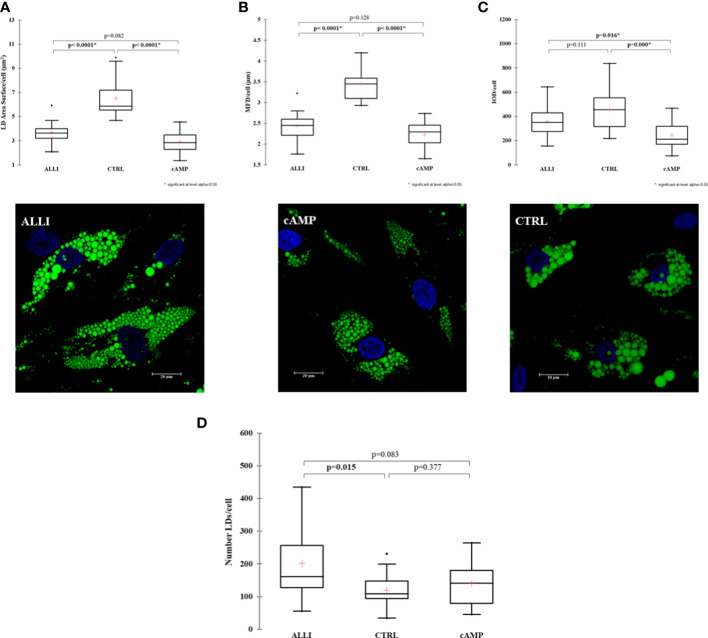
Results of lipid droplet analysis performed on SGBS cells. Boxplots show the median (horizontal lines), the first to third quartiles (box), and the most extreme values with the interquartile range (vertical lines). For all comparisons, differences between treatments on SGBS cells were statistically significant using the Kruskal-Wallis test and Bonferroni correction. **(A)** LD area per cell; **(B)** Maximum Feret Diameter (MFD) per cell; **(C)** Optical Density Intensity (IOD) per cell; **(D)** number of lipid droplets per cell in treated and CTRL cells.Representative confocal images of SGBS cells treated with allicin (ALLI), dibutyryl cAMP (cAMP), or control (CTRL) 6 days after differentiation and stained with BODIPY. Nuclear staining, DAPI. Images are representative of n. 15 biological replicates. ALLI, 12.5 µg/mL allicin-treated cells; CTRL, control cells; cAMP, 500 µM dibutyryl cAMP-treated cells.

Next, We investigated whether allicin has an effect on early adipogenic differentiation. Thus, we performed LD analysis in SGBS cells after ALLI treatment during the induction of adipogenesis. **Figure 1** shows statistically significant differences in area of LDs/cell, MFD/cell, and IOD/cell between treatments and illustrates Bodipy staining in SGBS cells after 6 days of treatment with allicin (ALLI), CTRL, and dibutyryl cAMP (cAMP). The area of LDs/cell was significantly lower in cells treated with cAMP and ALLI compared with cells from CTRL (*p <* 0.0001). No significant differences were observed between cAMP- and ALLI-treated cells (**Figure 2A**). A significant (*p <* 0.0001) decrease in MDF/cell was observed in cells treated with cAMP and ALLI compared with CTRL (*p <* 0.0001) (**Figure 2B**), while IOD/cell was significantly lower in cells treated with cAMP compared with cells treated with ALLI (*p* = 0.016) and CTRL (*p* = 0.0001) (**Figure 2C**). A significant increase in the number of LDs/cell (*p* = 0.015) was observed between ALLI treated cells and CTRL cells (**Figure 2D**).

### Allicin increases the number and area of small round mitochondria

Accurate analysis of mitochondria is critical for determining mitochondrial dynamics, so three different tools were used to characterize mitochondrial morphology. **Figure S2** shows the identification and classification of objects using adaptive thresholding (radius = 1.350 µm, C = 5) on images of ALLI- and cAMP-treated cells and CTRL. **Figures S2A, B** show the effects of the different treatments measured with the three tools, on the total number and area of mitochondria in SGBS cells. No statistical differences in mitochondrial distribution patterns were detected between treatments, although treatments with allicin and cAMP caused a shift toward a greater number of mitochondria (**Figure S2A**) and a greater total area of mitochondria compared with cells from CTRL (**Figure S2B**). These results suggest that ALLI treatment increases the number of mitochondria by inducing mitochondrial fission or biogenesis and thus also increases the total area of mitochondria.

**Figure 3:**
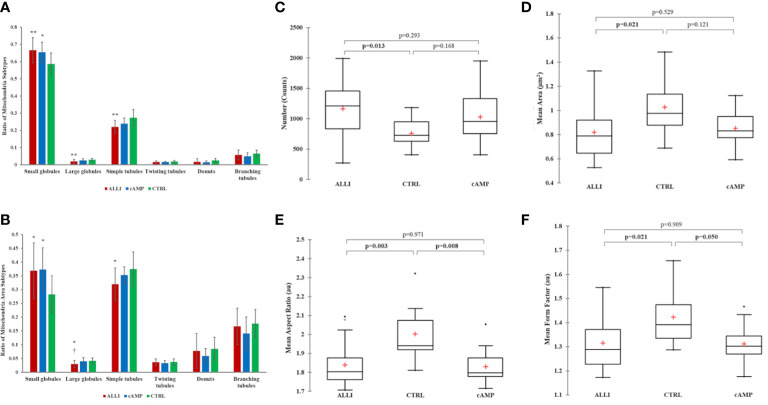
Mitochondrial analyses. Differences in the distribution of mitochondrial morphological characteristics were analysed between cells incubated with ALLI and cAMP compared with cells from CTRL. **(A)** Average ratio of mitochondrial subtypes within each treatment. The Kruskal-Wallis test was performed between ALLI-treated cells compared with CTRL (**p < 0.01) and cAMP-treated cells compared with CTRL (*p < 0.05). Differences between ALLI-treated cells and cAMP were also detected († *p* < 0.05). **(B)** The ratio of the surface area was significantly higher in ALLI- and cAMP-treated cells compared with CTRL by 30.3 and 31.7%, respectively (*p* < 0.05). The average ratio surface area of large globules showed a significant (* † *p* < 0.05) decrease in ALLI-treated cells by 24.9 and 28.6% compared with cAMP-treated and CTRL cells, respectively. Mitochondria features determined using the Mitochondria Analyser tool. **(C)** Number of mitochondria. **(D)** Mitochondrial size measured by mean area. **(E, F)** Mitochondrial shape measured by mean aspect ratio and mean form factor. Boxplots showed the difference between medians (horizontal lines), first to third quartiles (box), and the most extreme values within the interquartile range (vertical lines) between treatments. Statistical significance in the boxplots was determined by the Kruskal-Wallis statistical test with Bonferroni correction (*p <* 0.0167). ALLI, 12.5 µg/mL allicin-treated cells; CTRL, control cells; cAMP, 500 µM dibutyryl cAMP-treated cells.

Therefore, more than 26000 mitochondria were analysed using Micro2P software to classify six morphological subtypes and calculate the average proportion of mitochondrial subtypes within each treatment (**Figure 3A, B**). ALLI and cAMP treatments resulted in 13.6% (p < 0.01) and 11.5% (*p* < 0.05) more small globules, respectively, compared with CTRL. ALLI treatment reduced the percentage of mitochondria with large globules to 32.5% and that of the simple tube subtype to 19.5% compared with CTRL (*p* < 0.01) (**Figure 3A**). Accordingly, the ratio of surface area was significantly higher (*p* < 0.05) in ALLI- and cAMP-treated cells compared with CTRL at 30.3 and 31.7%, respectively, whereas the area of large globules in ALLI-treated cells decreased significantly (*p* < 0.05) (**Figure 3B**).

Specifically, the average ratio of mitochondrial amount in the different treatments was 53.4% in ALLI-treated cells, 28.6% in cAMP-treated cells, and 18.02% in CTRL. The relative percent area of total mitochondrial content between treatments was 51.9% in ALLI-treated cells, 27.8% in cAMP-treated cells, and 20.3% in cells from CTRL. Considering all treatments together, small globules (65.7%) and simple tubules (23.3%) were the most representative subtypes, followed by branched tubules (5.42%), large globules (2.3%), donuts (1.7%), and twisting tubules (1.6%). Accordingly, the percentage area of mitochondrial subtypes was 34.9% for small globules, 33.9.8% for simple tubules, 16.67% in branching tubules, 7.4% for donuts, 3.6% for large globules, and 3.5% for twisting tubules.

Mitochondrial Analyzer detected significantly higher numbers of mitochondria in ALLI-treated cells (**Figure 3C**), whereas the mean perimeter and area (**Figure 3D**) were significantly lower compared with cells from CTRL. No statistical differences were observed in the cells treated with cAMP. The shape of mitochondria, characterized by aspect ratio and form factor, decreased significantly under ALLI and cAMP compared with cells from CTRL (**Figures 3E, F**). Mitochondria Analyser tool was also used to quantify the morphological complexity of mitochondria. The mean length of branches and the total number of branches per mitochondrion were lowest in cells treated with ALLI and showed a significant difference in cells treated with cAMP (data not shown).

**Figure 4:**
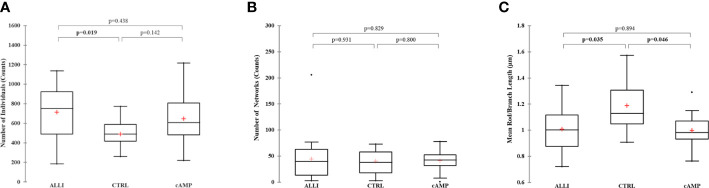
Summary statistics of mitochondrial network analysis performed with the MiNa tool on SGBS cells for each treatment. **(A)** Number of individual mitochondria. **(B)** Number of networks. **(C)** Mean value of rod/branch length. Boxplots show the median (horizontal lines), first through third quartiles (box), and the most extreme values with the interquartile range (vertical lines). The number of individual counts was statistically different in ALLI-treated SGBS cells from CTRL cells, using the Kruskal-Wallis test and Bonferroni correction (*p <* 0.0167). The number of networks showed no statistical difference. The mean length of rod/branch mitochondria significantly decreased in ALLI- (p = 0.035) and cAMP-treated cells (*p <* 0.046) compared with CTRL. ALLI, 12.5 µg/mL allicin-treated cells; CTRL, control cells; cAMP, 500 µM dibutyryl cAMP-treated cells.

Network parameters calculated with MiNa confirmed a significant (*p <* 0.05) increase in the number of individuals (puncta, rods and large) in ALLI-treated cells compared with CTRL cells (**Figure 4A**). The number of networks showed no significant differences between treatments (**Figure 4B**), but the mean value of rod/branch length significantly decreased in cells treated with ALLI (*p <* 0.05) and cAMP (*p <* 0.05) (**Figure 4C**).

### Mitochondrial analysis results classify treatments into non-overlapping groups

**Figure 5:**
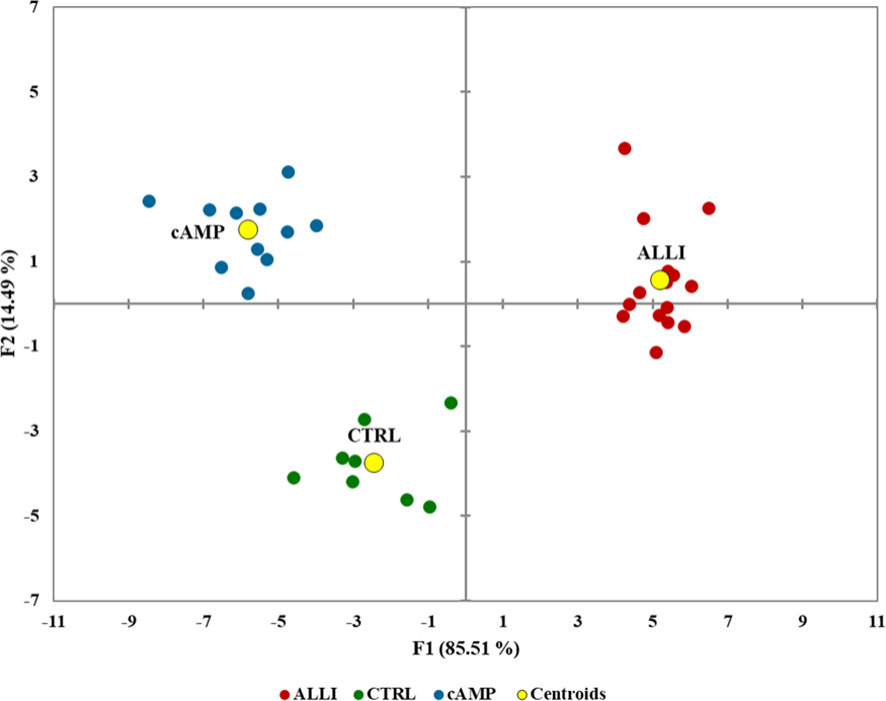
Biplot of canonical discriminant analysis to visualize the information of the whole mitochondrial dataset in the treatments and controls. F1 and F2 are canonical discriminant functions, and the two-component model separated the treatments. Centroids are shown as yellow circles. ALLI, 12.5 µg/mL allicin-treated cells; CTRL, control cells; cAMP, 500 µM dibutyryl cAMP-treated cells.

To determine the extent of treatment-specific variation in an unbiased manner, all Mitochondrial Analyzer measurements, six MiNa measurements from, and the percentage of the number and area of each mitochondrial subtype, number of objects, and total area calculated by Micro2P were integrated into a single multivariate model (DA) that maximized differences between treatments (**Figure 5**).

The model achieved clear separation of samples. With few exceptions, the treatments were grouped and divided into 3 clusters. Mitochondrial values of ALLI-treated cells were projected in the quadrant with a positive value for the F1 and F2 components, CTRL was projected in the quadrant with a negative value for F1 and a positive value for the F2 component. The projection of cAMP-treated cells was observed in the quadrant with negative value for F1 and positive value for F2. The significant morphological characteristics that distinguished the different experimental groups are shown in **Table 1**. This analysis showed that most of the variance between treatments in mitochondrial dynamics was based on elongation index, area of small globes, solidity of simple tubes, number of punctate and rod-shaped mitochondria (individuals), and number of simple tubes (**Table 1**). Discriminant functions were used to classify the treatments into the correct groups. To check the functionality and robustness of the classification model, a cross-validation test was performed, in which the success rate of correctly classified samples was 88.6%.

**Table 1:** Unidimensional test of equality of the means of the classes illustrating the contribution of different morphological features to the separation of differentially treated cells.

Variable	Wilk’s Lambda	F	p-value
Mean Aspect Ratio (au)	0.746	5.795	0.007
Simple tubules (%)	0.765	5.224	0.011
Mean Branch Length (µm)	0.779	4.817	0.014
Total Branch Length/area	0.789	4.533	0.018
Median Branch Length (µm)	0.802	4.205	0.023
Mean Network Size (Branches) (µm)	0.805	4.115	0.025
Mean Branch Length (µm)	0.808	4.050	0.026
Mean Form Factor (au)	0.815	3.863	0.031
Area_Large globules (%)	0.834	3.392	0.045
Mean Perimeter (µm)	0.834	3.381	0.046

The most discriminant parameters have a low Wilk’s lambda and a high F.

### RNAseq analysis

After removal of low abundance reads, the final mapping rate of the filtered transcript reads was 71.2%. Hierarchical clustering was performed on the initial analysis of the RNAseq results, that showed that the transcriptomic data was well-clustered according to the treatment. In addition, PCA showed the overall variability in the expression profile of the samples and treatments. Overall, there was a significant difference between CTRL and the treatments with cAMP and ALLI along the first principal component, which explained 61% of the variance, with smaller differences 28% along the second component (**Figure S3**).

Analysis of DEGs was performed between cells treated with allicin and cAMP compared to control cells and between allicin and cAMP treatments, based on a log_2_fold change of |1| and an FDR adjusted *p* value of ≤ 0.05.

iDEP951 expression analysis significantly identified (*p <* 0.05) 820 up regulated genes between cells treated with ALLI *vs* cAMP, 1417 up regulated genes between cells treated with ALLI *vs* CTRL and 1647 between cells treated with cAMP *vs* CTRL cells. Significantly down regulated (*p <* 0.05) genes were 640 between cells treated with ALLI compared with cAMP, 1085 genes between cells treated with ALLI *vs* CTRL and 1521 between cells treated with cAMP compared with CTRL cells (**Supplementary Table 1**).

### GO term and KEGG analysis enriches genes of treated cells in cellular respiration, mitochondrial organization, and thermogenesis

To further investigate the function of the DEGs, GO term enrichment analysis was performed. The up- and down regulated DEGs were significantly enriched in biological processes (BP), molecular functions (MF) and cellular components (CC).

In all comparisons, 30 BP, CC and MF GO terms were found to be significantly enriched (**Supplementary Tables 2**–**4**). Notably, BP terms such as ‘Oxoacid metabolic process’, ‘Small molecule metabolic process’, ‘Fatty acid metabolic process’, ‘Cellular respiration’, ‘Cellular lipid metabolic process’, ‘Energy derivation by oxidation of organic compounds’, ‘Monocarboxylic acid metabolic process’ were down regulated in the ALLI_cAMP comparison, but up regulated in the ALLI_CTRL and cAMP_ CTRL comparisons (**Supplementary Table 2**). Interestingly ‘Mitochondrion’, ‘Mitochondrial matrix’, ‘Mitochondrial inner membrane’, ‘Mitochondrial membrane’, ‘Mitochondrial envelope’, ‘Mitochondrial protein-containing complex’ CC terms were down regulated in ALLI_cAMP comparison, but up regulated in ALLI_CTRL and in cAMP_ CTRL comparisons (**Supplementary Table 3**).

In contrast, significantly enriched MF terms such as ‘Calcium ion binding’, ‘Cell adhesion molecule binding’, ‘Collagen binding’, ‘Extracellular matrix structural constituent’, ‘Glycosaminoglycan binding’, ‘Growth factor binding’, ‘Heparin binding’, ‘Integrin binding’ and ‘Signalling receptor binding’ were up regulated in the ALLI_cAMP comparison, but down regulated in the ALLI_CTRL and in the cAMP_CTRL comparisons (**Supplementary Table 4**). Interestingly, significantly enriched MF terms such as ‘Active transmembrane transporter activity’, ‘Electron transfer activity’, ‘Oxidoreductase activity’ and ‘Transmembrane transporter activity’ were up regulated in the ALLI_CTRL and in the cAMP_CTRL comparisons (**Supplementary Table 4**).

**Figure 6:**
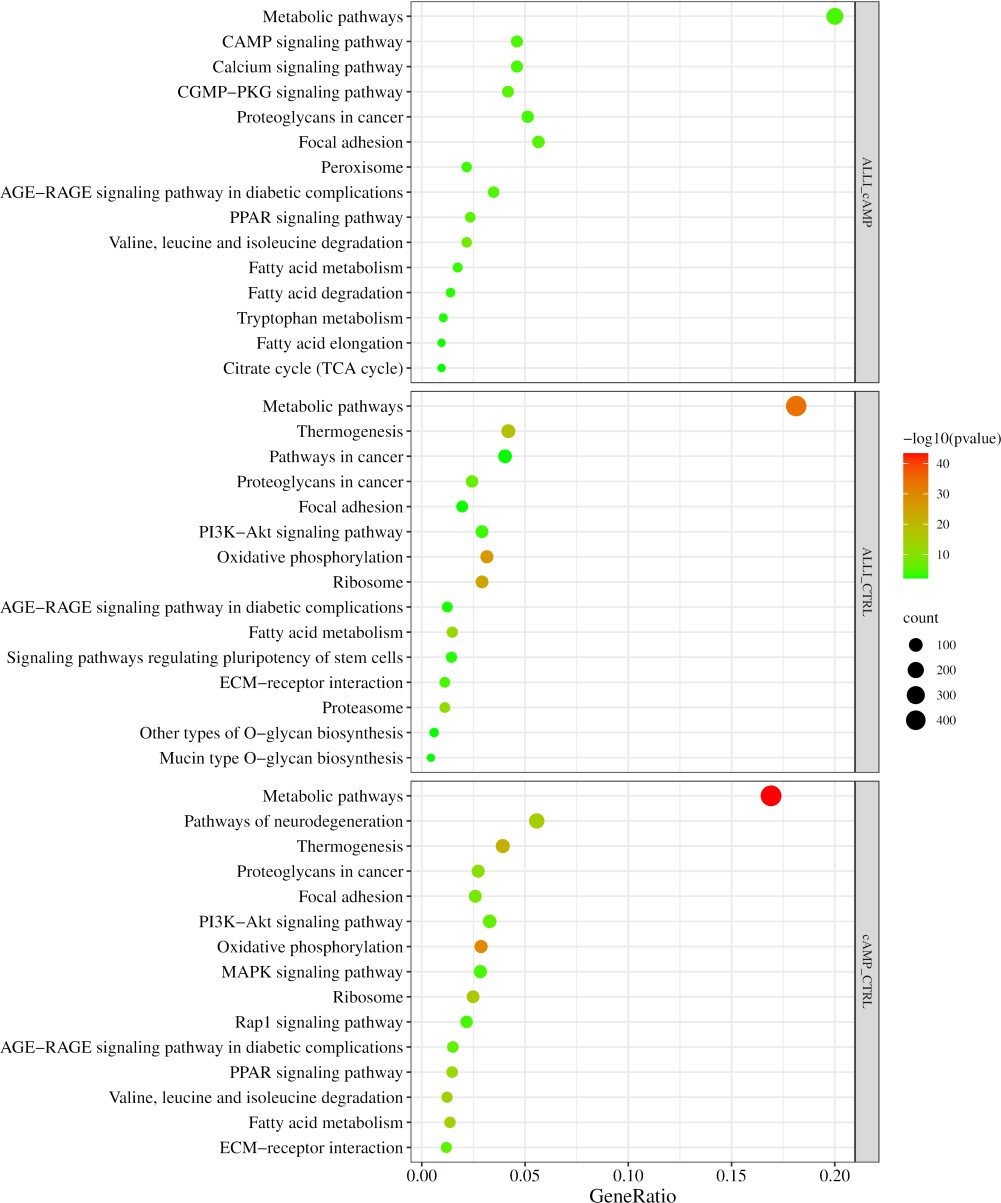
KEGG pathway enrichment analysis. Dot size represents the number of genes in each KEGG pathway; p.val (adjusted p-value): Red < orange < green. ALLI_cAMP = 12.5 µg/mL allicin-treated cells *vs* cAMP = 500 µM dibutyryl cAMP-treated cells; ALLI_CTRL = 12.5 µg/mL allicin-treated cells *vs* control cells; cAMP_CTRL =500 µM dibutyryl cAMP-treated cells *vs* control cells. Scatter plot was drawn by http://www.bioinformatics.com.cn/srplot.

**Figure 6** shows the results of the KEGG analysis in the form of a graphical representation of the scatter plots. Each figure shows the KEGG enrichment of 15 identified pathways for each treatment comparison with the corresponding GeneRatio, adjusted *p*-value, and number of enriched genes in the corresponding pathways. The GeneRatio is defined as the number of enriched candidate genes compared with the total number of annotated genes included in the corresponding pathway in the KEGG analysis. Therefore, a higher GeneRatio indicates a higher enrichment of candidate genes in the corresponding pathway. KEGG analysis showed that DEGs were significantly down regulated within the ‘PPAR pathway,’ Fatty acid metabolism, elongation and degradation, and in ‘Citrate cycle (TCA cycle)’ in ALLI_cAMP comparison. Of note, DEGs were over-expressed in pathways such as ‘cAMP signalling pathway,’ ‘Calcium signalling pathway,’ and ‘CGMP-PKG signalling pathway’ in ALLI_cAMP comparison (**Figure 6**). ALLI_CTRL and cAMP_CTRL had common DEGs significantly enriched in ‘Oxidative phosphorylation’ and ‘Thermogenesis’, whereas DEGs within the ‘PPAR signalling pathway’ were down regulated in ALLI *vs* cAMP and up regulated only in cells under cAMP treatment (**Figure 6**).

These results suggest that the browning effect of ALLI is only evident when compared with CTRL cells, so the ALLI-cAMP contrast was not discussed further.

### Construction of PPI networks and module analysis of DEGs in cells treated with allicin and positive control indicate brown adipocyte differentiation associated with an increase in AMPK and PPARA signalling through downregulation of extracellular matrix organization

The PPIs of all up regulated and down regulated DEGs with combined scores greater than 0.4 were constructed from the three comparisons, and each entire PPI network was analysed using Cytohubba. The ten most highly regulated hub genes with a high degree of connectivity between nodes are listed in **Table 2**. The highly regulated hub genes in the ALLI_CTRL and cAMP_CTRL comparisons shared 6 genes such as PPARG, FASN, SREBF1, SCD, PPARGC1A, and ACLY. Both comparisons, referring to CTRL, were similarly enriched in ‘Fatty acid synthase complex,’ ‘acetyl-CoA carboxylase complex,’ ‘AMPK signalling pathway,’ ‘PPARA activates gene expression,’ ‘Regulation of small molecule metabolic process,’ and ‘Thermogenesis’.

**Table 2:** Top 10 hub genes from up regulated DEGs calculated by Degree topological algorithm of Cytohubba plugin.

ALLI_CTRL			cAMP_CTRL		
ank	Gene	Score	Rank	Gene	Score
1	PPARG	100	1	FASN	100
2	IL1B	87	2	PPARG	97
3	FASN	86	3	CS	96
4	SREBF1	84	3	ACLY	96
5	EGF	78	5	SREBF1	94
5	SCD	78	6	SCD	89
7	APOE	74	7	PPARGC1A	87
7	PPARGC1A	74	8	ACADM	86
9	CD4	73	9	ACACA	79
10	ACLY	69	9	ACO2	79

ALLI_CTRL, 12.5 µg/mL allicin-treated cells vs control cells; cAMP_CTRL, 500 µM dibutyryl cAMP-treated cells vs control cells.

Four down regulated hub genes, such as FN1, THBS1, COL1A2, and CCN2, are common between ALLI- and cAMP-treated cells compared with CTRL cells (**Table 3**). Enrichment of both comparisons included ‘AGE-RAGE signalling pathway in diabetic complications’, ‘PI3K-Akt signalling pathway’, ‘Focal adhesion’, ‘ECM-receptor interaction’, and ‘TGF-beta signalling pathway’.

**Table 3:** Top 10 hub genes from down regulated DEGs calculated by Degree topological algorithm of Cytohubba plugin.

ALLI_CTRL			cAMP_CTRL		
Rank	Gene	Score	Rank	Gene	Score
1	FN1	96	1	FN1	179
2	MYC	61	2	IL6	130
3	CD34	45	3	CD44	104
4	THBS1	39	4	COL1A1	103
5	COL1A2	38	5	MMP2	99
5	THY1	38	6	ERBB2	83
5	HGF	38	7	THBS1	82
8	LOX	36	7	CCN2	82
8	DCN	36	7	CCND1	82
8	CCN2	36	10	COL1A2	79

ALLI_CTRL, 12.5 µg/mL allicin-treated cells vs control cells; cAMP_CTRL, 500 µM dibutyryl cAMP-treated cells vs control cells.

### Genes upregulated by allicin and cAMP are enriched in the target genes of AR and PPARG, which are involved in the positive regulation of cold-induced thermogenesis and fatty acid metabolism

TRED analysis (http://rulai.cshl.edu/TRED) allows to know interaction data between transcription factors (TFs) and the promoters of their target genes, including binding motifs (38). In the ALLI_cAMP comparison, the target genes of only 1 TF (c-MYC) were down regulated and 15 were up regulated; in the ALLI_CTRL comparison, 4 TFs were down regulated and 3 (AR, PPARA, PPAG) were up regulated; in the cAMP_CTRL, 15 were down regulated and the same 3 of the ALLI_CTRL comparison were up regulated (**Supplementary Table 5**).

The up regulated genes were enriched in the target of AR in all comparisons (14 genes), whereas genes enriched in the target of PPARG (37 genes) and PPARA (18 genes) were enriched only in the ALLI_CTRL and cAMP_ CTRL comparisons (**Supplementary Table 5**). The target genes of JUN, SP1, and TP53 were significantly down regulated in the ALLI_CTRL and cAMP_CTRL comparisons but up regulated in the ALLI_cAMP comparison (**Supplementary Table 5**). Down regulated DEGs were enriched in target genes of EGR1, ETS1, SMAD1, SMAD3, SMAD4, and TFAP2A in the cAMP_CTRL comparison, but up regulated in ALLI_cAMP (**Supplementary Table 5**).

Enrichment analysis of the 14 genes targeting to AR and common to all comparisons showed significant up-regulation of ‘Response to hormone, ‘Regulation of lipid metabolic process’, ‘Regulation of small molecule metabolic process’, ‘Cellular response to hormone stimulus’, ‘Zinc finger nuclear hormone receptor-type’ and ‘PPARA activates gene expression’. The MCODE plugin cluster analysis did not filter any cluster with a score ≥ 5 for these genes.

The 37 DEGs targeting AR that were common only between ALLI and cAMP treatments compared with CTRL cells and the 51 common DEGs targeting PPARG resulted in only one MCODE cluster. No cluster with a score ≥ 5 was found for common DEGs targeting PPARA.

**Figure 7:**
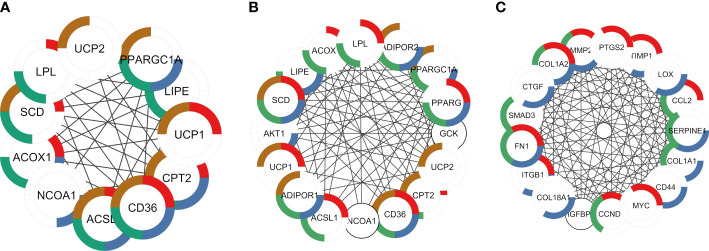
PPI networks identified by cluster functional analysis constructed with up- and down regulated DEG targets to TFs and overlapping to ALLI and cAMP treatments *vs* CTRL. The enriched pathways are marked in different colors. **(A)** Cluster 1 with a MCODE score of 7.83 achieved from up regulated genes target of AR. Red: genes enriched in ‘PPARG signalling pathway’ (FDR 1.82^-12^); brown: ‘Positive regulation of cold-induced thermogenesis’ (FDR 4.05^-10^); green: ‘Fatty acid metabolic process’ (FDR 3.08^-09^); and blue: ‘PPARA activates gene expression (FDR 4.59^-08^). **(B)** Cluster 1 with a MCODE score of 10.47 achieved from up regulated genes target of PPARG. Red: genes enriched in ‘PPARG signalling pathway’ (FDR 3.10^-13^); brown: ‘Positive regulation of cold-induced thermogenesis’ (FDR 4.02^-13^); green: ‘Fatty acid metabolic process’ (FDR 5.23^-13^); and blue: ‘AMPK signalling pathway’ (FDR 5.53^-12^). **(C)** Cluster 1 with a MCODE score of 13.67 achieved from down regulated genes target of SP1. Red: genes enriched in ‘Interleukin-4 and Interleukin-13 signalling (FDR 3.32^-13^); green: AGE-RAGE signalling pathway in diabetic complications (FDR 4.42^-12^); blue: ‘Extracellular matrix organization’ (FDR 7.12^-12^).

Enrichment analysis revealed the potential function of genes in each module. Shared DEGs targeted by AR and PPARG and over-expressed in ALLI_CTRL and cAMP_CTRL were enriched, among others, in ‘PPARG signalling pathway’ (**Figures 7A, B** red), ‘Positive regulation of cold-induced thermogenesis’ (**Figures 7A, B**, brown), ‘Fatty acid metabolic process’, ‘AMPK signalling pathway’ (**Figures 7A, B**, green), ‘AMPK signalling pathway’ (**Figure 7A**, blue) and ‘PPARA activates gene expression’ (**Figure 7B**, blue).

DEGs targeted by TP53, JUN and SP1 were down regulated in both ALLI_CTRL and cAMP_CTRL comparisons, but up regulated in ALLI_cAMP comparison. Shared DEGs targeted by SP1 and down-expressed in ALLI_CTRL and cAMP_CTRL were enriched in ‘Interleukin-4 and Interleukin-13 signalling’ (**Figure 7C**, red), and in ‘Extracellular matrix organization’ (**Figure 7C**, red). Interestingly, DEGs targeted by SP1 were found over-expressed in ALLI_cAMP comparison. Down regulated DEGs targeted by TP53 and JUN common to ALLI_CTRL and cAMP_CTRL were enriched in ‘Interleukin-4 and Interleukin-13 signalling’ and ‘IL-18 signalling pathway’ (data not shown).

### Allicin stimulation favors the differentiation into brown adipocyte

**Figure 8:**
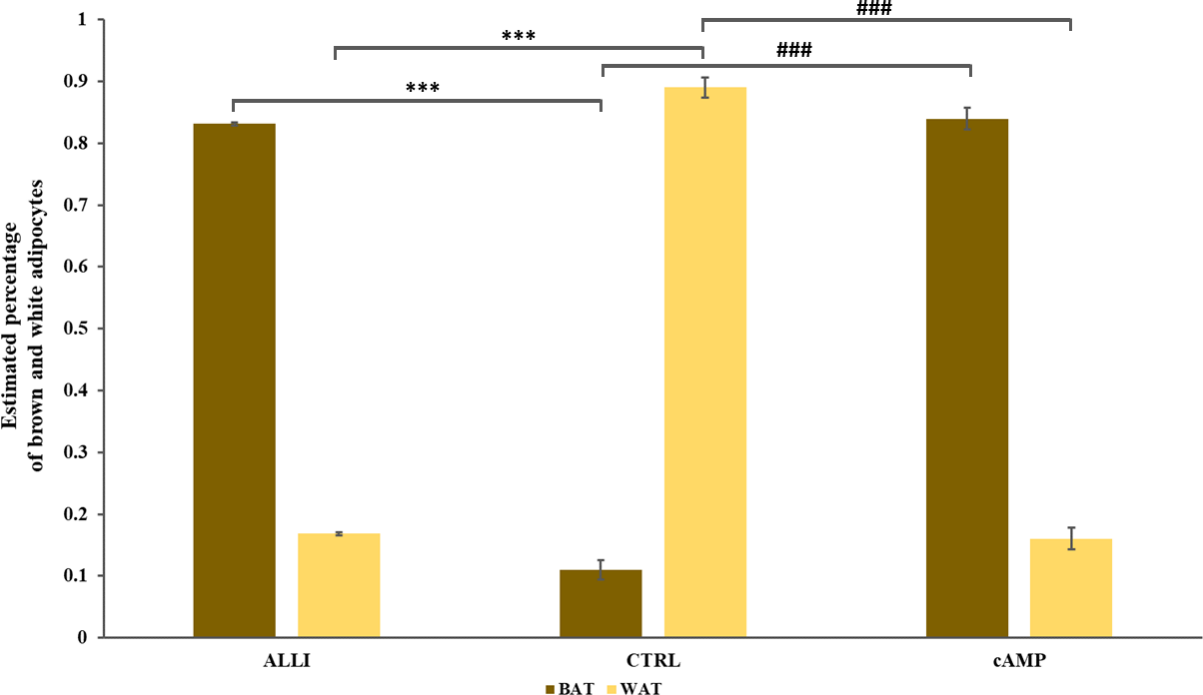
Statistical significance of PROFAT prediction percentage of brown and white adipocytes was determined using Euclidean distance and complete linkage on normalized gene expression values and analyzed by statistical test-t. *** = *p <* 0.0001 between estimated percentage of brown cells; ### = *p <* 0.0001 between estimated percentage of white cells. ALLI, 12.5 µg/mL allicin-treated cells; cAMP, 500 µM dibutyryl cAMP-treated cells; CTRL, control cells.

Allicin stimulation favors the differentiation into beige adipocyte The PROFAT tool (41) generated the heatmap of marker expression starting from normalized reads counts of SGBS cells. The estimation of BAT phenotype in ALLI- and cAMP-treated cells increased significantly (*p <* 0.0001) in comparison to CTRL cells. In contrast, WAT phenotype decreased significantly (*p <* 0.0001) in ALLI- and cAMP-treated cells compared with CTRL (**Figure 8**). These results evidenced that SGBS cells exhibit a gene expression pattern similar to that of brown cells during 6 days of differentiation under allicin treatment.

### Identification of common candidate targets among allicin, their organosulfur compounds and browning target genes

**Figure 9:**
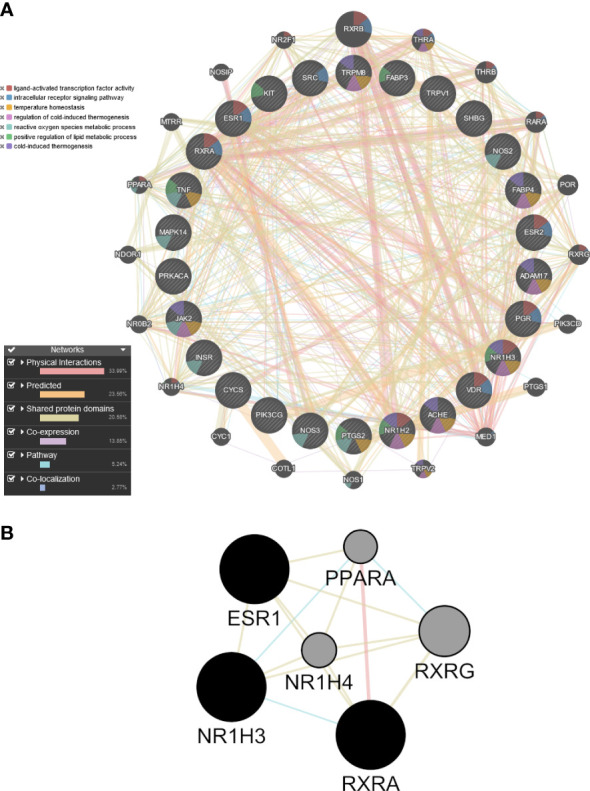
Preliminary PPI network constructed with GeneMANIA. **(A)** Twenty-six common targets between allicin, related fat-soluble organosulfur compounds and adipocytes-browning were built as assigned based on query gene. Genes are linked by functional associated networks filtered on their FDR score. Black nodes are the query targets and the larger the node, the higher degree of the node. The stronger interaction between node, the ticker and deeper colour of the edge. **(B)** Core PPI subnetwork generated by intersectional merge of PPI subnetworks according to the calculated degree, betweenness, closeness, eigenvector, LAC and network average values. ESR1, Estrogen Receptor 1; PPARA,Peroxisome Proliferator Activated Receptor Alpha; NR1H3, Nuclear Receptor Subfamily 1 Group H Member 3; NR1H4, Nuclear Receptor Subfamily 1 Group H Member 4; RXRA, Retinoid X Receptor Alpha; RXRG, Retinoid X Receptor Gamma.

Because allicin is rapidly converted *in vitro* to its related fat-soluble organosulfur compounds such as DATS, DADS and DAS, the potential targets of these compounds and of allicin were screened by computational target fishing from the PharmMapper, STITCH, Swiss Target Prediction and GeneCard databases. By overlapping the highest ranked common targets of allicin and related organosulfur compounds with the 315 overlapped ‘adipocyte-browning’ genes, 26 common targets between allicin compounds and adipocyte-browning were used to create a GeneMania network (**Supplementary Table 5**). The results of the analysis showed that these 26 targets correlated with 20 others and a total of 407 different links were predicted to construct a network linking these 46 genes (**Figure 9A**). The constructed network had 33.99% physical interactions and 23.56% predicted functional relationships between genes. In addiction 20.58% shared the same protein domain and 13.85% shared similar co-expression characteristics, other results were pathways (5.24%) and colocalization (2.77%) as shown in **Figure 9A**. The molecular functions of the top ranked targets, filtered by their FDR score, were reported as GO categories. The preliminary network illustrated that the genes, depicted by different colours in **Figure 9A**, were involved in ‘ligand-activated transcription factor activity’, ‘intracellular receptor signalling pathway’, ‘temperature homeostasis’, ‘regulation of cold induced thermogenesis’, ‘reactive oxygen species metabolic process’, ‘positive regulation of lipid metabolic process’, and ‘cold-induced thermogenesis’.

### PPI subnetwork construction and identification of core targets

A topological analysis of the preliminary network was performed using the CytoNCA plugin in Cytoscape to find the core proteins that form the preliminary network. The mean the degree (17.70), betweenness (48.96), closeness (0.49), eigenvector (0.106), LAC (12.40), network (10.95) values of the preliminary network was calculated, and the nodes of the preliminary PPI network that were above this mean were sorted out to build the corresponding subnetworks. Using the intersectional merge function in Cytoscape a core PPI subnetwork was extracted (**Figure 9B**) containing 6 key nodes (ESR1, NR1H3, NR1H4, PPARA, RXRA, RXRG) and 15 edges. Among these genes, Nuclear Receptor Subfamily 1 Group H Member 4 (NR1H4) and PPARA were significantly up regulated in the ALLI_CTRL comparison of SGBS cells (**Supplementary Table 1**), whereas estrogen receptor 1 (ESR1), Nuclear Receptor Subfamily 1 Group H Member 3 (NR1H3) and Retinoid X receptor alpha (RXRA) are common targets of allicin and related fat-soluble organosulfur compounds.

### The effects of allicin are related to mitochondrial biogenesis and lipid catabolism through the activation of core targets transcription factors

GO terms from biological process, cellular component, and molecular functions were examinedand the most enriched GO terms from biological process were ‘intracellular receptor signalling pathway’, ‘cellular response to lipid’, ‘hormone-mediated signalling pathway’, ‘response to steroid hormone’, ‘response to lipid’, whereas the most enriched GO terms from cellular component and molecular functions were ‘transcription regulator complex’, ‘nuclear receptor activity’, and ligand-activated transcription factor activity, respectively (data not shown).

KEGG pathways were analysed with a redundancy cut-off of 0.7, 17 pathways were statistically significant (FDR < 0.05) ‘PPAR signalling pathway’, ‘Adipocytokine signalling pathway’, ‘Thyroid hormone signalling pathway’, ‘Non-alcoholic fatty liver disease, ‘Insulin resistance’ and ‘Lipid and atherosclerosis’(data not shown).

**Figure 10:**
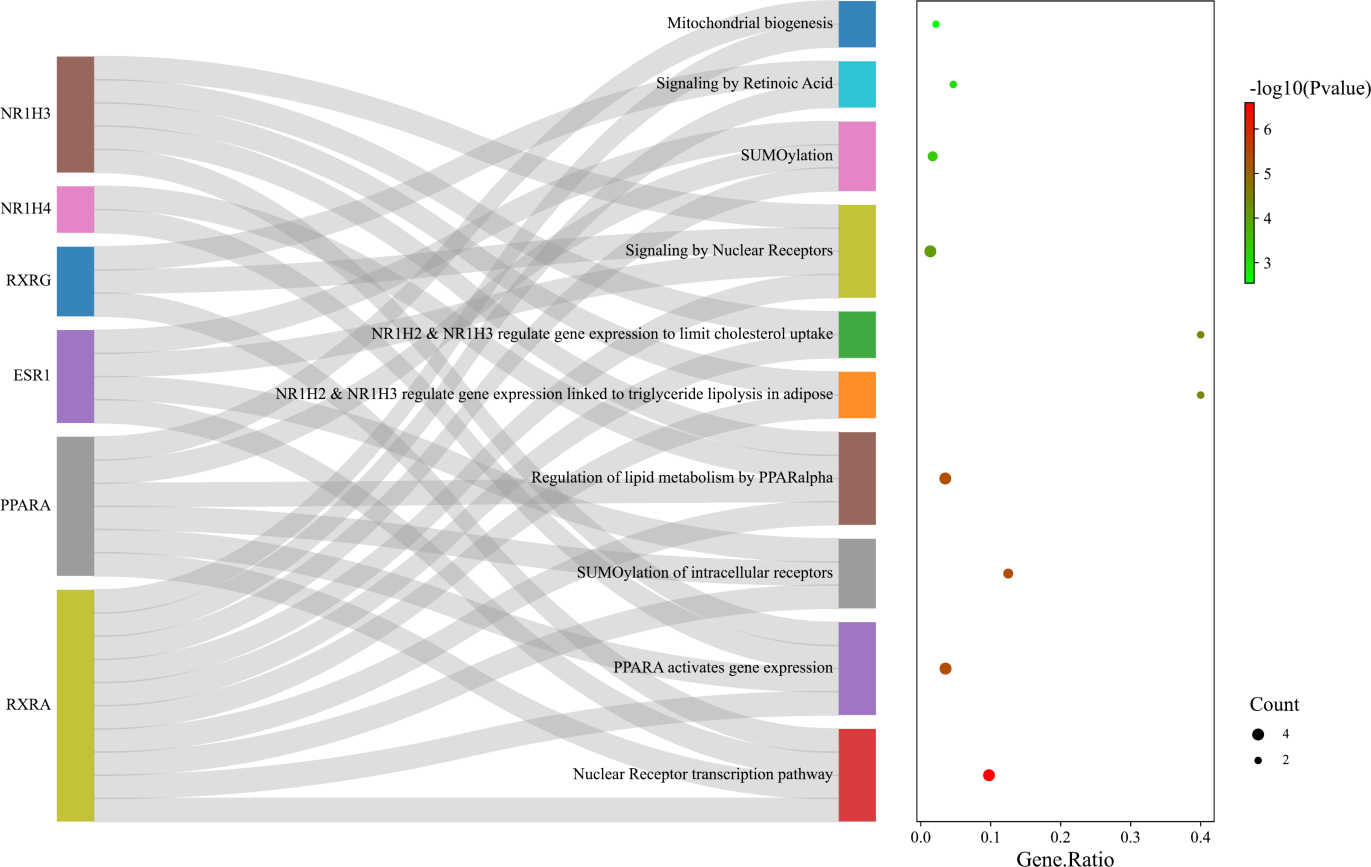
Bubble plot combined with Sankey diagram showing statistically significant Reactome pathways and the genes within each pathway. Figure was plotted by http://www.bioinformatics.com.cn/srplot.

The pathways enriched by Reactome analysis were ‘Nuclear Receptor transcription pathway’, ‘PPARA activates gene expression’, ‘SUMOylation of intracellular receptors’, Regulation of lipid metabolism by PPARalpha’ and ‘Mitochondrial biogenesis’, which were shown as bubble plot combined with a Sankey diagram (**Figure 10**).

In addition, the score of the 6 key nodes identified by the topological analysis was scored using VarElect. The score indicates the strength of the association between the target and the ‘cold induced thermogenesis’ phenotype (**Table 4**). ESR1, PPARA, NR1H3, and NR1H4 scored > 6.

**Table 4:** Score of the six key nodes evaluated by VarElect.

Gene Symbol	Description	-Log10(*P*)	Score
ESR1	Estrogen Receptor 1	2.35	14.91
PPARA	Peroxisome Proliferator Activated Receptor Alpha	2.07	11.56
NR1H3	Nuclear Receptor Subfamily 1 Group H Member 3	1.49	6.30
NR1H4	Nuclear Receptor Subfamily 1 Group H Member 4	1.63	6.09
RXRA	Retinoid X Receptor Alpha	1.44	5.89
RXRG	Retinoid X Receptor Gamma	0.55	0.56

## Discussion

As a thermogenic organ, BAT is known to enhance energy metabolism and weight loss (50), so promoting mass and activity of BAT is one of the most promising strategies against obesity. Treatment of white adipose cells with rosiglitazone or with other β-adrenergic agonists induces beige cells with similar properties as BAT (51). Induction of the beige/brown fat cell phenotype leads not only to thermogenesis, but also to lipolysis, which facilitates energy metabolism, and mitochondrial dynamics, which precede the depolarization associated with heat dissipation (23). A high rate of mitochondrial fragmentation and free fatty acid release promote mitochondrial uncoupling and energy expenditure (52). Knowledge of the signalling pathways that stimulate the transition from white to beige adipocytes, could help identify effective therapeutic strategies against obesity.

The discovery of functionally active BAT in adult humans and the possible recruitment of beige adipocytes by browning of WAT have introduced the way for new potential strategies for anti-obesity agents (53).

Previous studies have shown that SGBS cells gradually acquire BAT-like function in the absence of external stimulation during different days of differentiation, suggesting that lipid droplets t dynamics, and mitochondrial morphology (27) together with a differential expression of genes involved in extracellular matrix organization and oxidative stress are related to the brown fat phenotype (20). While it has been clearly demonstrated that the β3-adrenergic receptor (β3-AR) mediates thermogenesis in rodents (54), BAT is activated in humans by β2-AR signalling (55). Therefore, to bypass the ADRBs receptors dibutyryl-cAMP was chosen as a positive control.

The present study demonstrated that allicin supported the transition from white to beige adipocytes in SGBS after 6 days of differentiation and that the transformation of structural cell phenotype was evidenced by the dynamic changes in the size of LDs and the shape of mitochondria similar to those observed in the positive control.Lipolysis is generally considered an essential requirement for thermogenesis in brown and beige adipocytes, and any lipolytic compound could be a potential activator of thermogenesis (56). In HepG2 cells, allicin reduces lipid accumulation either by regulating AMPK-SREBPs and PKA-CREB signalling pathways (57) or by activating PPARA and FABP6 gene expression (58). The effect of allicin on lipid reduction argues for PPARγ/LXRα signalling in THP-1 macrophage foam cells (59). In the present work, ALLI and cAMP treatments decreased the area and diameter of LDs, but because the number of LDs/cell increased significantly with ALLI treatment, the lipolytic activity of allicin may have led to the formation of multilocular adipocytes, a feature of WAT browning. This is confirmed by the increased number of differentially expressed genes related to lipolysis, such as DNA fragmentation factor subunit alpha (DFFA), monoglyceride lipase (MGLL), perilipin 1 (PLIN1), patatin like phospholipase domain containing 2 (PNPLA2), lipoprotein lipase (LPL) and hormone-sensitive lipase (LIPE) in ALLI- and cAMP-treated cells (**Supplementary Table 1**). However, a thermogenic futile cycle of lipolysis/lipogenesis has been claimed to explain the unilocular to multilocular transformation during WAT browning (21). In 3T3-L1 cells exposed to β-adrenergic stimulation, remodelling of LDs involves first their reduction into small LDs and then their new formation and subsequent enlargement (21). Indeed, significant expression of negative regulators of lipolysis such as G0/G1 switch gene 2 (G0S2) and patatin like phospholipase domain containing 3 (PNPLA3) were also found in ALLI-treated SBGS cells as well as the mRNA levels of the perilipin 4 (PLIN4), diacylglycerol o-acyltransferase 1 (DGAT1), diacylglycerol o-acyltransferase 2 (DGAT2) and adipocyte glycerol transporter aquaporin7 (AQP7), (**Supplementary Table 1**), indicating that the cells store and export metabolites released during lipolysis. Moreover, other studies have shown that triglyceride lipolysis catalysed by PNPLA2 in mice brown adipocytes is not required to maintain body temperature during cold exposure (60, 61) and that other sources such as circulating glucose and fatty acids can balance thermogenesis (62).

During cold exposure mitochondrial reorganization and free fatty acid release synergize to facilitate uncoupling and thereby heat production (23). Concomitantly, mitochondria acquire a spheroid morphology driven by increased fission (63). Present results show an increased in number and area of mitochondria in cells treated with allicin, and the data was also confirmed by the reduction of elongation (mean aspect ratio) and by the change from round to filamentous shape (mean form factor) in ALLI- and cAMP-treated cells. Network parameters obtained by MiNa also show a significant decrease in mean rod/branch length in both treatments compare to CTRL cells. According to the Micro2P plugin, six different subtypes of mitochondria with the highest proportion of small globules were classified in ALLI- and cAMP-treated cells. Canonical DA further evidenced that mitochondrial parameters specifically those related to mean aspect ratio, percentage of simple tubules, mean branch length, accurately clustered differentially treated cells and CTRL cells.

The high content of organosulfur compounds in garlic suggests that many of its active compounds may have anti-adipogenic effects by promoting the expression of genes specific for brown adipocytes (64). Recent data showed that allicin promotes browning of 3T3-L1 mouse adipocytes and iWAT by inducing the expression of brown marker genes through KLF15 signalling (16) or through the SIRT1-PGC1α-TFAM pathway (17).

PCA analysis based on reads clearly grouped the data set on the first component between CTRL cells and cells treated with ALLI or cAMP. On the second component, the treatments are separated, but the replicates of the same point were very close, indicating robust reproducibility of the data.

Classical thermogenesis is activated by adrenoreceptors that promote cAMP synthesis for PKA activation and expression of downstream targets (65). Intracellular cAMP levels are maintained by a balance between the rate of synthesis mediated by adenylate cyclase and the rate of degradation regulated by cAMP phosphodiesterase 3 (PDE3). Allicin is known to increase intracellular cAMP by inhibiting phosphodiesterase activity in isolated human platelets (66, 67) or by increasing adenylate cyclase activity in the human bronchial epithelial cell line (68). In adipose tissue, PDE3 inhibitors increase intracellular cAMP levels, thereby enhancing lipolysis (69). The present results showed a significant up-regulation of PDE3B in ALLI_CTRL and cAMP_CTRL (**Supplementary Table 1**), resulting in an increase in intracellular cAMP and downstream genes involved in lipolysis, such as LIPE and PLIN1, and in browning, such as TBX1 and UCP1 (**Supplementary Table 1**). This is consistent with the results obtained in adipose tissue of mice fed a high-fat diet when treated with cilostazol, a selective inhibitor of phosphodiesterase III with multiple effects on metabolism (70). In addition, cilostazol, which has antiplatelet, antithrombotic, and vasodilatory properties similar to allicin, increased the intracellular concentration of cAMP, which stimulated the expression of thermogenic and brown specific genes (70). The BP GO terms enrichment, such as cellular respiration and cellar lipid metabolic process as well as CC GO terms related to mitochondria were significantly up regulated in ALLI- and cAMP-treated cells compared with CTRL cells, suggesting similar activity in cells with different treatments, but was opposite when ALLI-treated cells were compared with cAMP-treated cells. MF GO terms, such as oxidoreductase activity, were up regulated in ALLI- and cAMP-treated cells compared with CTRL cells, but down regulated in ALLI-treated and cAMP-treated cells. Therefore, the positive browning effect of ALLI treatment was evident only in comparison with CTRL cells, but not in comparison with cAMP incubation. However, ALLI and cAMP treatments shared the most up regulated hub genes such as PPARG, FASN, SREBF1, SCD, PPARGC1A, and ACLY, which are related to fatty acid metabolic process, fatty acid oxidation and response to cold. The lowest down regulated hub genes common to ALLI and cAMP treatments FN1, THBS, COL1A2 and CCN2 were enriched in ECM receptor interactions, integrin cell surface interactions and focal adhesion. This is consistent with the down regulation of collagen, integrin and laminin genes (COL1A1, COL1A2, ITGA2, ITGA3, ITGA4, ITGA5, LAMA1, LAMA2, LAMA3; **Supplementary Table 1**) observed in SGBS cells during differentiation (20) demonstrating their ability to adjust cytoskeletal reorganization according to their size, LDs dynamics and thermogenesis (71).

KEGG pathway enrichment confirmed that oxidative phosphorylation, thermogenesis, and fatty acid metabolism were the most significantly up-regulated pathways in the ALLI_CTRL and cAMP_CTRL comparisons, whereas ECM-receptor interaction, PI3K-Akt signalling pathway and Focal adhesion were downregulated. In contrast, pathways related to PPAR and fatty acid metabolism were significantly downregulated in the ALLI_ cAMP comparison.

Interestingly, an *in vivo* study suggests that the allyl-containing sulphides of garlic significantly enhance thermogenesis and increase epinephrine and norepinephrine levels in rat plasma (72), which is why allicin may interact with the adrenergic receptor (AR), which is one of the most effective mechanisms to deplete excess energy through cAMP/PKA-dependent signal transduction (73). In the present study, up regulated DEGs common to ALLI_CTRL and cAMP_CTRL comparisons and the targets of transcription factor AR were significantly associated with ‘PPARG signalling pathway’, ‘positive regulation of cold-induced thermogenesis’, ‘fatty acid metabolic process’ and ‘PPARA activates gene expression’. All of these metabolic pathways and processes share the genes for fatty acid translocase (FATP or CD36), acyl-coA synthetase long chain family member 1 (ACSL1) and carnitine palmitoyltransferase II (CPT2), each of which is involved in the storage and recycling of fatty acids, their conversion to acyl-CoA and transport to mitochondria (74). Their co-expression is clearly part of the thermogenesis programme. In 3T3-L1 adipocytes, CD36 has been found to play an important lipolytic role (75) and its translocation from the cell membrane to lipid droplets mediates the release of long-chain fatty acids by exocytosis (76). In human macrophages aged garlic extract inhibits CD36 expression through modulation of the PPARγ pathway (77), but in SGBS cells, its over expression together with that of LPL, and aquaporin 7 (AQP7) (**Supplementary Table 1**) can lead to triglycerides uptake and then lipolysis associated with heat production (78). In addition, CD36 has been found to be a scavenger receptor required for coenzyme Q (CoQ10) uptake in BAT and therefore essential for adaptive thermogenesis and BAT morphology, (79). Of note, CoQ10 is up regulated in ALLI-treated SGBS cells (**Supplementary Table 1**).

ACSL1 and CPT2 have been shown to be required for fatty acid oxidation for cold-induced thermogenesis (80). Interestingly, all of these genes are downstream targets of the nuclear transcription factor PPARA, which is expressed in metabolically active tissues such as brown adipose tissue (81). In contrast, down regulated DEGs targets of SP1 and other TFs, such as TP53 and JUN, were involved in ‘Interleukin-4 and Interleukin-13 signalling’ and ‘Extracellular matrix organization’, ‘Cytokine-mediated signalling pathways’ and ‘IL-18 signalling pathways’, as previously described in SGBS cells (20). In particular, the down regulation of fibronectin (FN1), collagen type I alpha 1 chain (COL1A1), collagen type I alpha 2 chain (COL1A2) is associated with that of the zinc finger transcription factor early growth response-1 (EGR1) (**Supplementary Table 1**) and, in mice, with a concomitant increase of beige cells differentiation and a decrease in genes encoding the extracellular matrix proteins (82). The down regulations of cell-surface glycoprotein CD44 and its receptor ONP in SGBS cells treated with allicin and cAMP (**Supplementary Table 1**) further confirms the browning activity of the compounds present in garlic. CD44 was recently recognized as a major receptor for an extracellular matrix component that plays an essential role in promoting obesity and diabetes (83).

Brown features were also confirmed by PROFAT analysis, which revealed a significant increase in 80% of genes related to BAT phenotype.

Using open-source tools, computational target fishing facilitates the investigation of biological targets of bioactive molecules using the reverse pharmacophore mapping approach (84) (**Supplementary Table 6**). To understand potential targets involved in the browning process six major targets ESR1, NR1H3, RXRA, PPARA, NR1H4, and RXRG were extracted from the comparison of targets of allicin and related organosulfur compounds with browning genesafter topological analysis. The targets were strongly associated, and enrichment analysis confirmed the involvement of these genes in limiting cholesterol uptake, lipolysis and mitochondrial biogenesis, all processes in which allicin plays a role. The lipolytic role of allicin may be related to the activation of PPARA through the release of fatty acids. RXRA forms heterodimers with PPARA to regulate the expression of genes involved in fatty acid oxidation (ACOX1, ACADM, CYP4A1, HMGCS2), fatty acid transport (CD36, SLC27A1, CPT2), and lipid storage (PLIN) (85), that were over expressed by ALLI treatment (**Supplementary Table 1**). This is consistent with the activation of PPARA promoted by allicin in palmitic acid-loaded HepG2 cells (58). Again, garlic essential oil significantly up regulated PPARA expression level in the liver of HFD-fed mice compared with control mice (86). Moreover, PPARA was found to be associated with the expression of superoxide dismutase (CuZn-SOD) in human aortic endothelial cells (87), a scavenger of ROS, which is consistent with the antioxidant properties of allicin. Of note, ESR1 is known to induce a selective beiging in 3T3-L1 cells leading to ATGL-mediated lipolysis (88). Moreover, in human and mouse adipocytes ESR1 promotes mitochondrial remodelling and thermogenesis through uncoupled respiration by regulating the mitochondrial gene POLG1 (89). All of downstream genes of these metabolic pathways, such as SOD1, ATGL, and POLG were significantly expressed in SGBS cells (**Supplementary Table 1**).

## Conclusion

Overall, this study supports the modulatory role of allicin in stimulating the brown phenotype of SGBS cells, which is associated with an increase in mitochondrial biogenesis and lipid catabolism. The possible mechanism of this interesting process may be based on the partial interaction of allicin within the regulatory steps of cAMP signalling and PPARA signalling.

However, the study has some limitations, because neither down-regulation of SIRT5 nor significant up-regulation of KLF15, as recently reported, was detected in SGBS cells. The mechanism by which allicin promotes browning and induces mitochondrial biogenesis is not yet fully elucidated, and functional studies could be performed to further investigate the browning effect.

## Data availability statement

The original contributions presented in the study are included in the article/**Supplementary Material**. Further inquiries can be directed to the corresponding author.

## Author contributions

UA and MC designed the study. UA performed the experiments and collected data. MC collected data measurements, performed statistical analyses prepared the figures and wrote the manuscript. MW and DT provided SGBS cells and were involved in the revision of the paper. All authors read and approved the manuscript. All authors contributed to the article and approved the submitted version.
