# Supplementary figures and images for: Effects of allicin on human Simpson-Golabi-Behmel syndrome cells in mediating browning phenotype
Source: Front Endocrinol (Lausanne). 2023 Mar 1;14:1141303. doi: 10.3389/fendo.2023.1141303 (PMC10014806; doi:10.3389/fendo.2023.1141303)

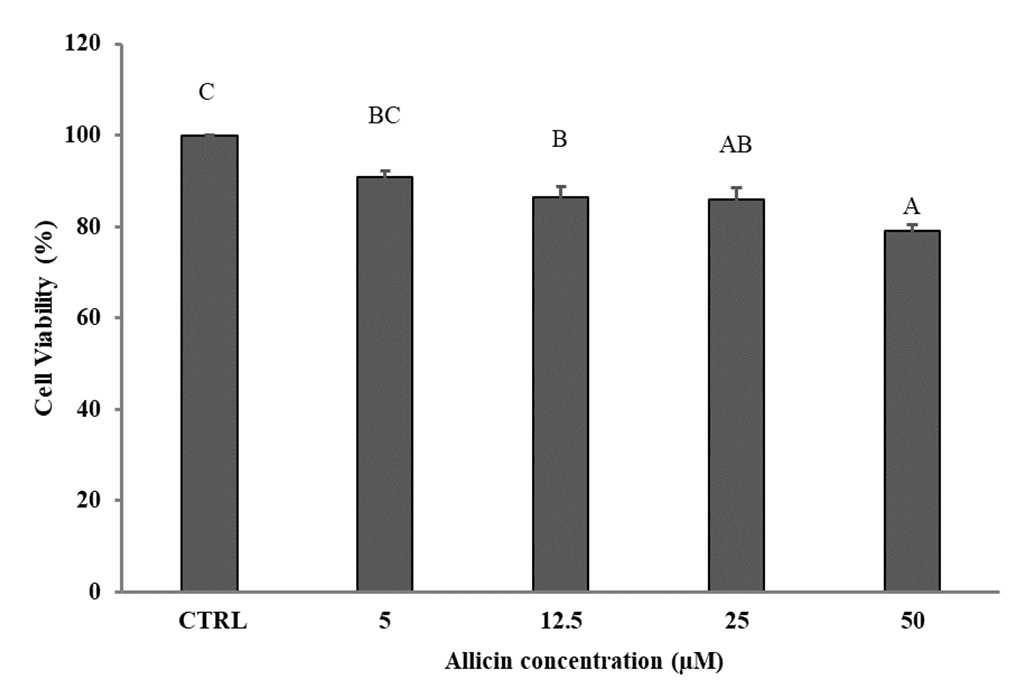

Supplement: Supplementary file 1 [file Image_1.tif]

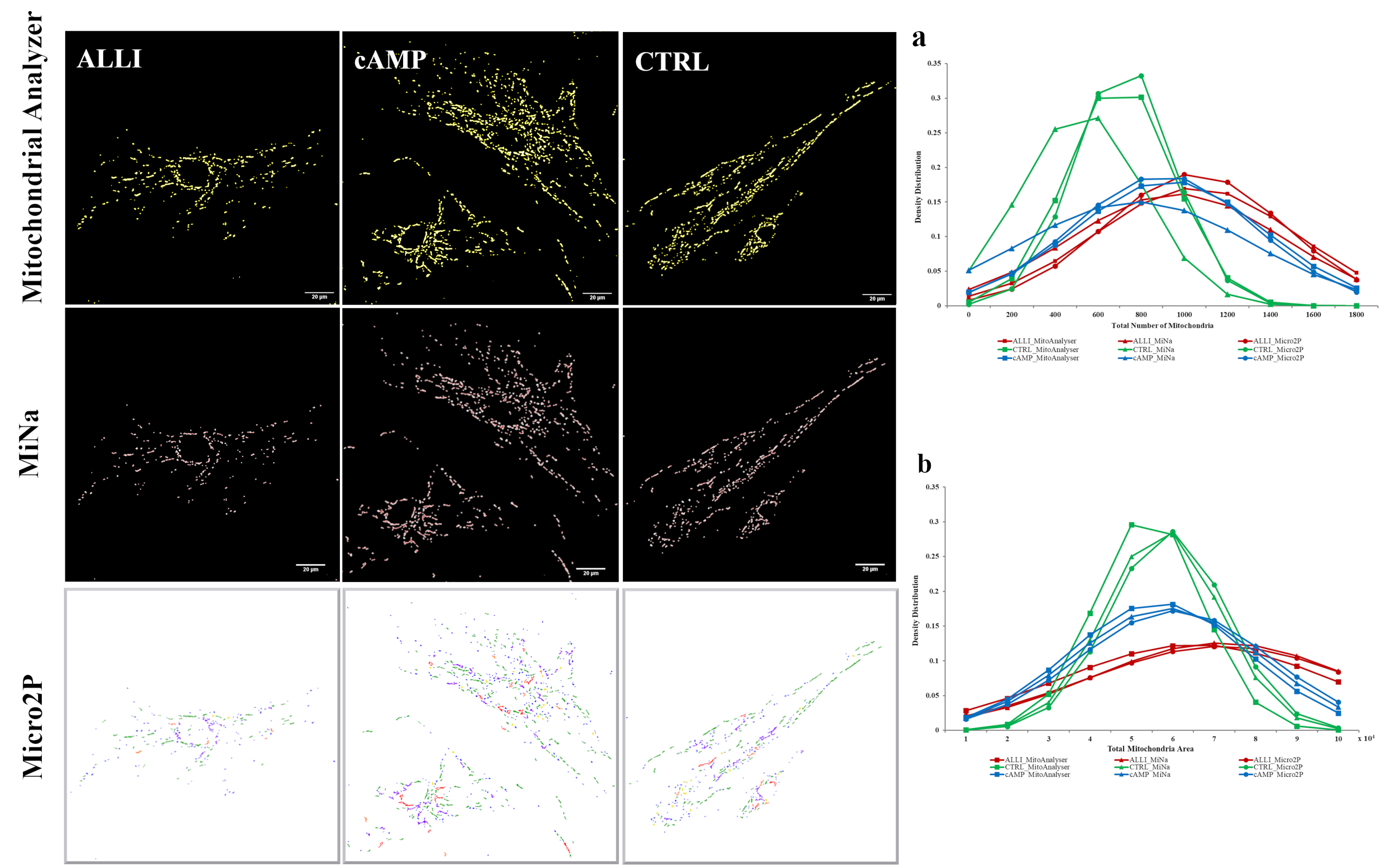

Supplement: Supplementary file 2 [file Image_2.tif]

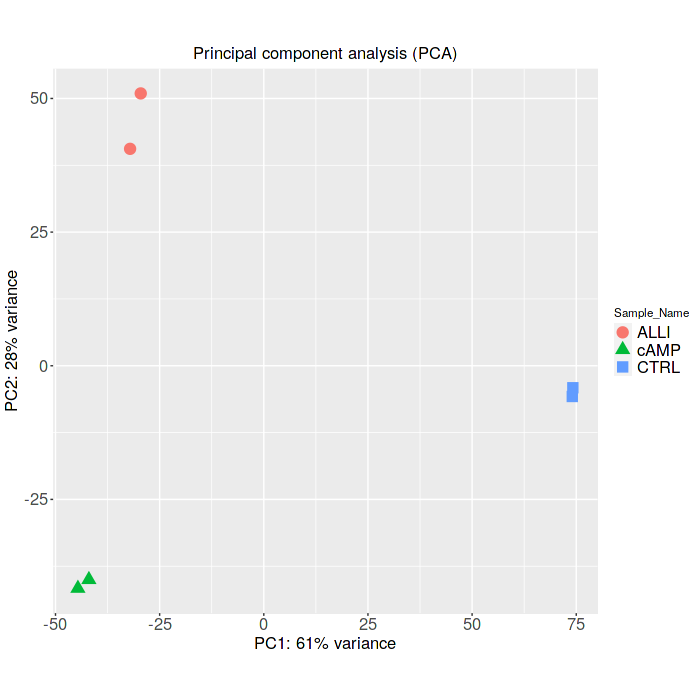

Supplement: Supplementary file 3 [file Image_3.tif]
